# Outcomes of Team-Based Digital Monitoring of Patients With Multiple Chronic Conditions: Semiparametric Event Study

**DOI:** 10.2196/75170

**Published:** 2025-12-08

**Authors:** Ross Graham, Itzik Fadlon, Parag Agnihotri, Christopher A Longhurst, Ming Tai-Seale

**Affiliations:** 1 Department of Sociology, University of California San Diego, La Jolla, CA, United States; 2 Department of Economics, University of California, San Diego, La Jolla, CA, United States; 3 Department of Medicine, University of California San Diego, La Jolla, CA, United States; 4 Department of Pediatrics, University of California San Diego, La Jolla, CA, United States; 5 Department of Bioinformatics, University of California San Diego Medical Center, La Jolla, CA, United States; 6 Department of Family Medicine, University of California San Diego, 9500 Gilman Drive, La Jolla, CA, 92093, United States, 1 6508155485

**Keywords:** remote patient monitoring, telemedicine, hypertension, digital medicine, multiple chronic conditions

## Abstract

**Background:**

Remote patient monitoring (RPM) has emerged as an effective strategy for controlling hypertension by enabling patients to collect and transmit blood pressure (BP) data outside the clinic and supporting proactive care team interventions. While its benefits for hypertension management are well established, less is known about its effectiveness in patients with multiple chronic conditions (MCC), who experience higher morbidity, mortality, and costs.

**Objective:**

This study aimed to evaluate the impact of an electronic health record (EHR)–integrated, team-based RPM program on patients with hypertension, alone or co-occurring with ischemic heart disease, type 2 diabetes, or both. This study aimed to determine whether referral to the program was associated with reductions in systolic blood pressure (SBP) across these patient groups.

**Methods:**

We analyzed EHR data from patients referred by their primary care physicians to the University of California San Diego Health’s Digital Health Program between October 2020 and July 2022. Eligible patients had hypertension, either alone or accompanied by at least 1 coexisting condition, such as ischemic heart disease or type 2 diabetes. Participants received a Bluetooth-enabled BP cuff and ongoing support from a multidisciplinary team, including nurse care managers and a pharmacist. A semiparametric event study design was used to estimate changes in SBP over 24 months, comparing prereferral and postreferral outcomes. To understand the program’s impact, outcomes were analyzed for the full cohort of all referred patients and then scaled to reflect the average change in SBP among the program participants.

**Results:**

Among patients who had been referred to the program, those with hypertension only experienced an average reduction of 9.70 (SE 0.80) mm Hg in SBP by the end of the analysis horizon of 1 year. Patients with hypertension and either diabetes or ischemic heart disease experienced a reduction of 6.61 (SE 1.12) mm Hg, and those with all 3 conditions experienced a reduction of 6.60 (SE 1.72) mm Hg. The average reductions in SBP among active participants were 16.83 mm Hg, 13.22 mm Hg, and 16.01 mm Hg, respectively.

**Conclusions:**

A team-based, EHR-integrated RPM program was associated with clinically meaningful SBP reductions among patients with MCC. The program leveraged existing EHR workflows for referral and monitoring and provided technical and clinical support to patients. These findings suggest that EHR-integrated RPM services can achieve substantial improvements in BP in high-risk populations. As reimbursement for RPM expands, such models represent a promising strategy for addressing hypertension and the disproportionate burden of MCC at the population level.

## Introduction

### Hypertension and Multiple Chronic Conditions

Hypertension is a widespread chronic disease affecting at least 33% of people aged 30 to 79 years worldwide [1] and approximately 48% of US adults [2], and contributes to poor cardiovascular, metabolic, mental health, and other chronic conditions. Approximately 27% of US adults have multiple chronic conditions (MCC) [3]. This patient population comprises up to 80% of Medicare costs [4], despite being the least studied among individuals with chronic diseases [5], and requires the most overall health care [6]. Common co-occurring chronic conditions include hypertension, type 2 diabetes, and ischemic heart disease. Among Medicare beneficiaries with MCC, diabetes and hypertension were the fourth most common combination (29.6% of MCC patients) in men aged <65 years and the fifth most common combination in men and women aged >65 years (28.6% and 27.5%, respectively) [7]. Ischemic heart disease and hypertension were the fifth most common combination in men aged <65 years (24.6%) and the third most common combination in men aged >65 years (39.0%) [7]. In men, the triad of ischemic heart disease, hypertension, and diabetes was the fourth most common [7]. Another study of US adults found that co-occurring diabetes and hypertension was the second most likely combination of MCC in all men and women aged >44 years [8]. Hypertension, ischemic heart disease, and diabetes share common pathophysiological processes. Their co-occurrence contributes to an increased risk of cardiovascular events and all-cause mortality [9,10].

### Remote Patient Monitoring

Remote patient monitoring (RPM) programs have shown positive effects in controlling chronic diseases such as hypertension and hyperlipidemia, proving feasible and effective in improving clinical outcomes in diverse patient populations [11-14]. These programs leverage technologies to empower patients and health care providers with regular, consistent collection of clinical data within everyday contexts for patients. They increase patient confidence and agency in managing chronic diseases and lower clinical exacerbations and hospitalizations, costs, and travel inconvenience for patients [15,16]. However, few health delivery organizations have scaled these solutions broadly, often citing the start-up investments involved and the need to pivot toward improved IT infrastructure [16], as well as complications that arise from new billing procedures and regulations around data privacy [17]. Some physicians and patients have expressed concern about the technological requirements of RPM, either with respect to patient capacity to use the technology consistently and effectively or that the technology itself might not be reliable over a sustained period [15]. However, reviews suggest that in practice, these concerns are often unfounded [17]. Even patients with English as their second language adapt well to RPM when provided with multilingual tools for assistance [18,19]. Generally, combining RPM with nurse or counselor follow-up and structured self-management interventions, such as patient education sessions, increases its effectiveness [20]. RPM is mostly used for cardiovascular health management [21] but is also effective in other domains, for example, remote glucose monitoring for diabetes [22].

### RPM in Patients With Hypertension and MCC

RPM of blood pressure (BP) for hypertension care shows evidence of sustainable decreases in patient systolic blood pressure (SBP) [12,23]. It reduces instances of white coat hypertension diagnosis by relying on real-world, at-home readings as opposed to office visits [24]. RPM effectively aids rural and medically underserved individuals and could play an important role in bridging the care gaps in chronically underserved regions [25], as well as racial minorities [26]. However, RPM has been previously shown to lead to stronger clinical improvements among racial, ethnic, and geographic groups who already experience better clinical outcomes (eg, White patients and those who live in affluent localities) due to lower RPM adoption by Black or Hispanic patients and those who live in less advantaged communities [27,28]. These effects are magnified when skills-based education of patients and community clinical providers generally serving medically underserved populations accompanies the technical rollout of RPM [29]. RPM has effectively helped control BP in postpartum women with hypertension [30].

RPM is effective in reducing the clinical burden of patients with MCC. Clinical trials and real-world observational and cohort studies show that RPM can lower mortality risk and SBP among patients with MCC who have heart disease and hypertension [24,31]. An RPM trial of patients with hypertension and diabetes observed a 9.1-point SBP reduction, bringing 51% of participants under 130/80 mm Hg within a year [32]; another observed an 11-point SBP reduction over 4 months [33]. A recent prospective observational study of patients with hypertension and high cholesterol, offering RPM alongside a patient support system, yielded a 9.7-point SBP reduction after 12 months [14].

Therefore, this study aimed to evaluate the impact of an electronic health record (EHR)-integrated, team-based RPM program on SBP among patients with hypertension, with a focus on patients with MCC.

## Methods

### The RPM Program

Hypertension management has been a priority at the University of California (UC) San Diego Health as part of a broader effort to mitigate cardiovascular risk among patients. The RPM service is a team-based, EHR-integrated service at the UC San Diego Health for patients with poorly managed hypertension. The program aims to lower cardiovascular risk while also reducing patient burden in managing their health and has been provided to a broad and diverse population of patients. Primary care physicians (PCPs) were introduced to the digital health service provided by the Population Health Services Organization via a presentation, either at departmental meetings or at a primary care retreat in 2021. They were shown how to refer eligible patients—those with a BP trend ≥140/90 mm Hg—within the EHR under a Care Coordination tab. PCPs were encouraged to refer patients as part of established practice guidelines as a complement to their existing care practices. Once referred by their PCP, patients who were interested in participating completed a short questionnaire with the Digital Health team to confirm that their smartphone supported the app used to record BP measurements. This questionnaire ascertained that their digital literacy was sufficient to take daily BP readings by confirming that they were comfortable downloading and setting up a smartphone app and using Bluetooth. Eligible participating patients were then provided with iHealth Ease or Omron free of charge, both of which are Bluetooth-enabled digital BP meters that automatically transmit data to the patient portal of the EHR via a Health Insurance Portability and Accountability Act (HIPAA)-compliant smartphone app.

**Figure 1.**
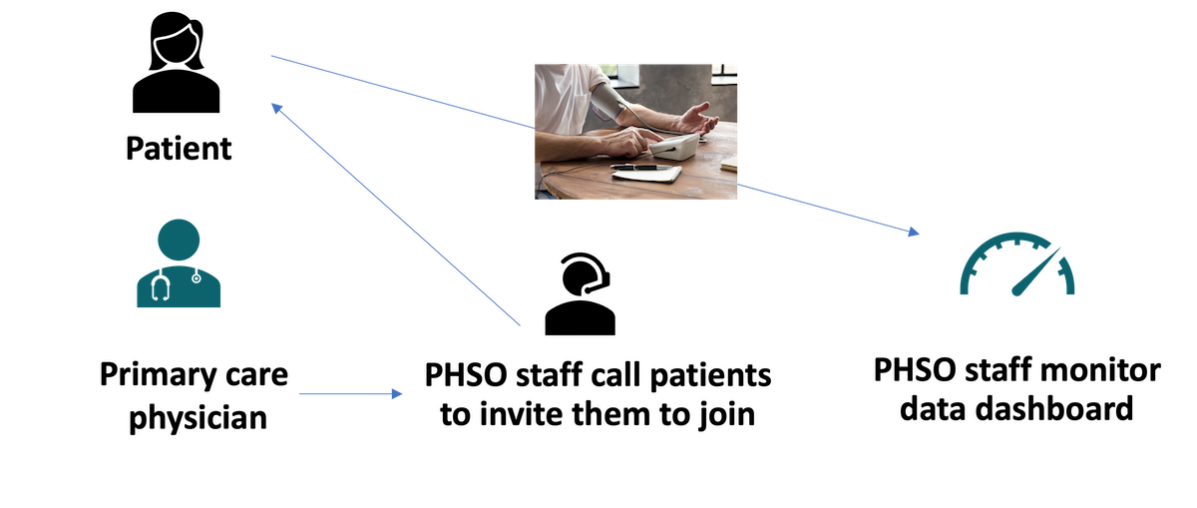
Outline of the intervention workflow. PHSO: Population Health Services Organization.

Enrollees are supported by a digital health specialist via telephone or, in cases where patients have extensive problems, via a visit to the patient’s home for device setup and any technical issues. These measures attempt to mitigate equity issues arising from variations in technological literacy, which is crucial given that the target population tends to be older. The EHR system stratifies BP measures into 3 risk groups: normal, high or priority, and critical, and presents them on a daily dashboard. Nurse care managers and a pharmacist review the dashboard on a daily basis and make treat-to-target adjustments per protocol, encompassing medication adjustments and behavioral change recommendations. Participating patients were advised to take BP measurements daily and were contacted if they did not submit a reading at least once every 30 days. Patients were advised to participate in the program for at least 6 months and had technical support available for the entirety of their participation (Multimedia Appendix 1) [27]. This ongoing outreach incentivizes adherence, where integration of monitoring into digital technology minimizes the clinical burden while expanding patient inclusion and furthering equity. Outlined in Figure 1, the program workflow is initiated with the PCP referring to the patient via the EHR. A digital health specialist then calls the patient to onboard them, enabling the patient to transmit BP measurements that are then monitored by the Population Health Services Organization staff using a central dashboard.

### Sample  

Our study sample was drawn from a subset of PCP referrals that occurred between October 2020 and July 2022 (n=2512). Patients were excluded if they were nonambulatory, under 18 years of age, pregnant, institutionalized, or dependent on supplemental oxygen. The analysis focused on a subsample of referred patients with MCC. Referral timing—the intervention point—varied across the study window. To assess the program’s longer-term impact rather than short-term reductions in SBP that might result from program initiation, we included SBP observations from months 7 to 12 after referral. This yielded 2206 patients with both prereferral and postreferral data for empirical analysis. Baseline data included prior BP readings, sex, age, and address from the EHR, along with self-reported race and ethnicity. Patient addresses were linked to the Healthy Places Index [34], which aggregates neighborhood-level indicators across California—such as education, employment, income, and housing—to approximate local socioeconomic conditions.

### Data Analysis

We used a semiparametric event study to assess the program’s impact on SBP among patients with hypertension, focusing on those with co-occurring type 2 diabetes or ischemic heart disease, as well as those with all 3 conditions simultaneously. Our event study implementation assesses the effects of the “event” of being referred to the digital health program based on deviations of patient outcomes from prereferral trends. This design makes causal inference more complex and limited compared to a randomized clinical trial but allows us to analyze a real-world care program within a large health care system. We provide estimates for all referred patients in the event study analysis in the 2 steps described subsequently.

In the first step, we adjusted the raw outcome variables based on preintervention data using a linear time trend in calendar months and age fixed effects to account for underlying trends in patients’ BP that would have occurred in the absence of the intervention. We used data from 12 to 2 months before the referral event as the baseline period. Month 1 before referral was excluded to remove possible transitory changes preceding the referral. We then computed residuals, namely, the differences between observed SBP values in our sample and the values predicted by our model as if the intervention had not occurred.

In the second step, we examined the change in the residualized outcomes between the preevent analysis period (months 12 to 1 before the referral) to the postevent analysis period (months 7 to 12 following the referral). This analysis identified changes in SBP around the time of referral, detecting deviations from the outcome predicted by our model of patients with hypertension described in step 1, thereby indicating the impact of the program on SBP. The analysis used regressions that included patient fixed effects, which accounted for time-invariant heterogeneity in outcomes across patients. Overall, we used the comparison of SBP outcomes before and after the intervention relative to the underlying trend as the estimates for the impact of referral on BP outcomes. Significance threshold was *P*<.05.

### Ethical Considerations

This quality improvement project was deemed nonhuman subject research and exempted from institutional review board review by the UC San Diego Health Aligning and Coordinating Quality Improvement, Research, and Evaluation Committee. No compensation was provided to participants in exchange for their participation. Data were stored on encrypted UC San Diego Health servers in accordance with Health Insurance Portability and Accountability Act and UC San Diego Health policies.

## Results

Table 1 provides average characteristics for our study’s population age, sex, race or ethnicity, and Healthy Places Index of residence.

**Table 1.** Sample characteristics (N=2206).

	Hypertension only (n=1318)	Hypertension with either diabetes or ischemic heart disease (not both) (n=745)	Hypertension with both diabetes and ischemic heart disease (n=143)
Age (y), mean (SD)	63.2 (15.2)	67.1 (12.8)	69.6 (11.5)
Proportion of patients aged ≥65	0.54	0.65	0.69
HPI^a^ percentile, mean (SD)	64 (25)	60 (26)	59 (27)
Female, n (%)	716 (54.32)	422 (56.64)	73 (51.05)
Race or ethnicity, n (%)			
Non-Hispanic White	805 (61.08)	343 (46.04)	66 (46.15)
Black or Hispanic	267 (20.26)	225 (30.20)	39 (27.27)

a HPI: Healthy Places Index.

Table 2 has 3 parts. Part A presents the estimates from the full referred population. Observations from before the referral come from months −12 to −1, and observations from after the referral come from months 7 to 12. Robust SEs are clustered at the patient level. Baseline levels are reported as subpopulation means of the outcome variable in month −1 relative to referral. The results indicated notable reductions in SBP in all subpopulations. Patients with hypertension only experienced a reduction of 9.758 (SE 0.81; *P<*.001) mm Hg. For those with hypertension and either diabetes or ischemic heart disease (but not both), the reduction was 6.599 (SE 1.13; *P*<.001) mm Hg. Patients with all 3 conditions—hypertension, diabetes, and ischemic heart disease—showed a reduction of 6.604 (SE 1.73; *P*<.001) mm Hg. Baseline SBP levels at the time of referral (month t=−1) were 138.4 mm Hg for the hypertension-only group, 131.4 mm Hg for those with co-occurring hypertension and either diabetes or ischemic heart disease, and 128.82 mm Hg for those with all 3 chronic conditions.

**Table 2.** Program’s impact on systolic blood pressure across patient groups.

	Hypertension only	Hypertension with either diabetes or ischemic heart disease (not both)	Hypertension with both diabetes and ischemic heart disease
Part A^a^^,^^b^			
Treatment effect on referred group, mean (SE)^c^	−9.758 (0.81)^c^	−6.599 (1.13)^c^	−6.604 (1.73)^c^
Baseline SBP, mean (SD)^d^	138.4 (21.4)	131.4 (23.3)	128.8 (22)
Part B^e^^,f^			
Initial take-up rate (SE)^c^	0.5797 (0.0136)^c^	0.4993 (0.0183)^c^	0.4126 (0.0413)^c^
Part C			
Treatment effect on SBP scaled by take-up rate	−16.83	−13.22	−16.01

a Difference between hypertension only and hypertension with either diabetes or ischemic heart disease (not both) columns: 3.16 (SE 1.39; *P*<.05).

b Difference between hypertension with either diabetes or ischemic heart disease (not both) and hypertension with both diabetes and ischemic heart disease columns: −0.006 (SE 2.06).

c *P*<.001

d SBP: systolic blood pressure.

e Difference between hypertension only and hypertension with either diabetes or ischemic heart disease (not both) columns: −0.0803 (SE 0.0227; *P*<.01).

f Difference between hypertension with either diabetes or ischemic heart disease (not both) and hypertension with both diabetes and ischemic heart disease columns: −0.0867 (SE 0.0456; *P*<.1 ).

Part B reports the initial take-up rates of the program, which describes the number of enrollees who completed at least 1 electronic BP reading. This rate varied significantly across the patient groups. The initial take-up rate for the hypertension-only group was 57.97 percentage points (pp; SE 1.36 pp, *P*<.001). For those with hypertension and either diabetes or ischemic heart disease, the take-up rate was 49.93 pp (SE 1.83 pp, *P*<.001). Patients with all 3 conditions had a take-up rate of 41.26 pp (SE 4.13 pp, *P*<.001). The difference in initial take-up rates between those with hypertension only and those with co-occurring combinations (hypertension and either diabetes or ischemic heart disease) was −8.03 pp (SE 2.27 pp, *P*<.001), and between those with co-occurring combinations and those with all 3 conditions was −8.67 pp (SE 4.56 pp, *P=*<.057).

Part C presents the average treatment effect among participants, which was derived by dividing the treatment effect estimates from Part A by the take-up rates from Part B, thereby estimating the treatment effect for patients who enrolled in the RPM. Among program participants, the average treatment effect was a SBP reduction of 16.83 mm Hg for the hypertension-only group, 13.22 mm Hg for those with hypertension and either diabetes or ischemic heart disease, and 16.01 mm Hg for those with all 3 chronic conditions. These results indicate that the digital health program effectively reduced SBP in patients with hypertension and MCC.

## Discussion

### Principal Findings

The study results demonstrate that the team-based RPM service substantially improved BP management for patients with hypertension. This positive impact was especially notable among those with MCC, specifically patients with co-occurring diabetes or ischemic heart disease, or all 3 conditions simultaneously [7,8,35]. Although the MCC groups had a lower baseline SBP—potentially indicating more intensive prior management—this study still substantiates and extends the findings of previous prospective quality improvement programs [14] and randomized controlled trials focused on patients with hypertension alongside hyperlipidemia, diabetes [32,33], or heart disease [24]. This shows the value of RPM for managing chronic conditions in the context of a robust care team and technical support for patients who tend to be older and are more likely to have multiple conditions beyond hypertension. Indeed, there is in situ evidence that with sufficient care team and technical support, age is not a limiting factor in RPM adherence for hypertension management if patient intention to control their hypertension remains high [36]. The observed reductions in SBP were similar to those reported by comparable programs embedded within large academic health centers, which typically include ongoing care team support, multilingual care, and extensive technical resources [19]. Therefore, our findings illustrate the capacity of RPM initiatives to improve care quality.

### Implications

Concrete clinical findings such as these call for increased uptake of RPM. Most states now provide Medicaid reimbursement for RPM [21], and as the financial incentives of RPM become more apparent—1 recent RPM program for cardiovascular health saved 173% of the program cost [37]—we expect that health systems will increasingly turn to RPM programs for improved clinical outcomes. We expect the financial benefits of RPM to increase as more health centers adopt similar programs and benefit from economies of scale and accrued knowledge about best practices for establishing such programs, as well as managing patient support procedures from a technical and clinical perspective. Our EHR-integrated team-based RPM service leverages the primary care physician-patient relationship, EHR infrastructure, and population health nurse care managers to improve clinical outcomes among patients with hypertension and MCC. It also leverages strong technical support resources from a large academic medical center. Findings from this real-world implementation of evidence bode well for further dissemination. Furthermore, reservations about patient capacity to operate technology are swiftly being addressed through the development of increasingly passive, discrete wearable sensors, such as rings [38]. Further technological developments promise an increasingly frictionless deployment of RPM programs.

### Limitations

This study has several limitations. First, while patient fixed effects were included to control for time-invariant heterogeneity, unmeasured confounders could have influenced the results. Second, for ethical reasons, the study eschewed an experimental design using randomization, as this would prevent patients randomized into a control group from receiving optimal care as recommended by their PCP. Although this represents a methodological limitation, it mirrors real-world conditions in which embedding RPM into routine care is an organizational priority, given the existing evidence supporting team-based practice assisted by RPM [39]. However, it is possible that patients referred to the program during the study period might differ systematically from those who were not in some unanticipated way. Third, the generalizability of findings from active participants is restricted to that group, as it is possible that they are more motivated, more comfortable with using digital technology, or healthier. However, findings based on all referred patients may be more broadly generalizable to referred individuals. Fourth, patient adherence varied across patient groups, which affects the ongoing effectiveness of the RPM program in addressing health disparities and equity. It is plausible that sicker patients may be less likely to adhere due to the complex tasks of managing their health. Finally, the digital health program was implemented as a team-based care model supported by technology. Importantly, this study does not attribute the observed reduction in BP solely to the technological component of RPM.

### Conclusions

This study contributes to the growing literature emphasizing that EHR-integrated RPM with a team-based management approach is associated with better clinical outcomes among patients with uncontrolled hypertension and MCC. As health systems adopt more value-based care models, programs such as these can leverage the broad adoption of EHRs, nurse care managers working alongside PCPs, and the proliferation of digital RPM devices to drive better population-level outcomes. The lower adherence rates among patients with MCC highlight a clear mandate for RPM technology development; solutions must offer maximal ease of use to effectively accommodate the complexity of managing MCC.

## Supplementary material

10.2196/75170 Multimedia Appendix 1 Population Health Services Organization digital health protocol for outreach and ongoing care.

## Abbreviations

BP: blood pressure
EHR: electronic health record
MCC: multiple chronic condition
PCP: primary care physician
RPM: remote patient monitoring
SBP: systolic blood pressure
pp: percentage points
