# Supplementary material for: Outcomes of Team-Based Digital Monitoring of Patients With Multiple Chronic Conditions: Semiparametric Event Study
Source: JMIR Cardio. 2025 Dec 8;9:e75170. doi: 10.2196/75170 (PMC12685231; doi:10.2196/75170)
Supplement: Multimedia Appendix 1 [file cardio-v9-e75170-s001.docx]

**Appendix: Population Health Team Digital Health Protocol for Outreach and Ongoing Care**

This appendix provides the exact digital health team outreach protocol. It is designed based on the Milliman Care Guidelines (more details can be found at <https://www.mcg.com/>).

Subjective
@NAME@ is a @AGE@ @SEX@ who I have contacted regarding: {phtoutreach:33397} related to the Digital Health Program for {rpmtask:32434} Monitoring. Was able to establish telephonic contact with the patient and verified patient’s identity with full name and date of birth.

Patient’s most recent Remote Patient Monitoring (RPM) {rpmtask:32434} reading of: *** with the following device(s): {digitalequipment:29472}

Contact outcome: {phtoutcome:33398}

Service Type Updated: {rpmyesnona:32440}

Motivational interviewing and positive affirmation techniques deployed with an emphasis on health coaching:

Blood Pressure Discussion:
-How to take BP Accurately (AHA guidelines): Patient well rested, arm at heart level, confirm cuff placement/fit; both feet flat on ground; bladder empty, prior to caffeine or activity. For any unusual readings / asymptomatic, rest/reposition/recheck - {rpmyesnona:32440}
-Frequency (at least x1/week, consistent times or as otherwise directed by MD):{rpmyesnona:32440}
-Patient is taking BP medications as prescribed? {rpmyesnona:32440}

-Patient has questions regarding BP management / medication (routing for potential f/u) {phtrns:33399}

Diet:
-HTN Patient following DASH diet: {rpmyesnona:32440}
-DM Patient following low glycemic diet: {rpmyesnona:32440}
-Patient requires additional dietary support {rpmrefer:32437}

Exercise:
-Patient regularly exercises: {rpmyesnona:32440}
-Patient requires additional exercise support {rpmrefer:32437}

RPM Disclaimer:
Reiterated that RPM f/u is available M-F 8:30a-4:30p (with the exception of National/University Holidays), does not replace emergent care, as there may be a delay in the upload/analysis of your blood pressure/blood sugar readings.

In the event of a Hypertensive Crisis (BP > 180 + symptoms of CP, Severe HA/Blurred Vision, SOB) or Hyperglycemic Medical Emergency (Blood Sugar > 300 + symptoms of Frequent Urination, N/V Increased Thirst and/or Hunger) please contact 911 or go to your nearest ED.

Plan:
Direct contact information provided and patient encouraged to reach out with further questions or concerns. DH Team to continue to monitor peripherally via Digital Health Dashboard or Self-Report outreach. Routing to {phtrns:33399} as FYI.
